# Targeted prebiotic application of gluconic acid-containing oligosaccharides promotes *Faecalibacterium* growth through microbial cross-feeding networks

**DOI:** 10.1093/ismejo/wraf027

**Published:** 2025-02-12

**Authors:** Hiroki Negishi, Ayumi Ichikawa, Saori Takahashi, Hiroshi Kano, Seiya Makino

**Affiliations:** Wellness Science Labs, Meiji Holdings Co., Ltd., Tokyo 192-0919, Japan; Wellness Science Labs, Meiji Holdings Co., Ltd., Tokyo 192-0919, Japan; Wellness Science Labs, Meiji Holdings Co., Ltd., Tokyo 192-0919, Japan; Wellness Science Labs, Meiji Holdings Co., Ltd., Tokyo 192-0919, Japan; Wellness Science Labs, Meiji Holdings Co., Ltd., Tokyo 192-0919, Japan

**Keywords:** gut microbiota, prebiotics, *Faecalibacterium*, *Parabacteroides*, cross-feeding, maltobionic acid

## Abstract

The gut microbiome plays a crucial role in human health, and certain bacterial species, such as *Faecalibacterium prausnitzii*, are particularly beneficial. This study conducted a comprehensive investigation of prebiotic compounds that showed potential for specifically promoting beneficial gut bacteria. Using in vitro fecal cultures and a human intervention study, we identified maltobionic acid and lactobionic acid as compounds that specifically promoted *Faecalibacterium* growth both in vitro and *in vivo* without significantly affecting *Bifidobacterium*, which is typically increased by traditional prebiotics. In a human intervention study (n = 27), a significant increase was observed in *Faecalibacterium* abundance following maltobionic acid supplementation, with effectiveness correlating with the initial *Parabacteroides* abundance. Mechanistic investigations revealed a cross-feeding pathway between gut bacteria. In this pathway, *Parabacteroides* species converted the gluconic acid moiety of maltobionic and lactobionic acids to glucuronic acid, which was then preferentially utilized by *Faecalibacterium*. These findings suggest that gluconic acid-containing oligosaccharides are promising prebiotics for the targeted enhancement of beneficial *Faecalibacterium* and underscore the importance of microbial interactions in prebiotic research, offering new avenues for personalized microbiome modulation strategies.

## Introduction

The human gut microbiome plays a pivotal role in maintaining host health, with certain bacterial species significantly contributing to various beneficial effects. *Faecalibacterium prausnitzii* has emerged as a key player due to its potential protective effects against inflammatory diseases and its association with overall gut health [1, 2]. This species produces butyrate, a short-chain fatty acid that possesses anti-inflammatory properties and is crucial for the maintenance of colonic health [3]. Reduced abundance of *F. prausnitzii* has been linked to various conditions, including inflammatory bowel disease, colorectal cancer, and type 2 diabetes, underscoring its importance in maintaining gut homeostasis [4–6]. Animal studies have demonstrated that *F. prausnitzii* administration prevents physiological damage in chronic low-grade inflammation models and improves metabolic parameters in high-fat diet-fed mice [7]. These findings highlight the potential therapeutic value of increasing *F. prausnitzii* abundance in the gut.

The extreme oxygen sensitivity of *F. prausnitzii* limits its direct use as a probiotic [8], prompting researchers to explore alternative strategies to harness its health-promoting properties. One approach involves the use of prebiotics. However, studies on the effects of traditional prebiotics on *F. prausnitzii* have often been limited or inconsistent. For instance, although inulin-type fructans and galactooligosaccharides consistently increase the abundance of *Bifidobacterium* populations, their effects on *F. prausnitzii* are variable [9–12]. This inconsistency may be attributed to the competition between different bacterial species, highlighting the need for more targeted prebiotic approaches.

Complex interactions between different bacterial species in the gut, particularly cross-feeding mechanisms, play crucial roles in shaping microbial communities [13]. However, our understanding of these interactions in relation to prebiotic metabolism is limited, hindering the development of targeted strategies to enhance the growth of beneficial bacteria beyond *Bifidobacterium*.

Recent efforts to modulate gut microbiota have highlighted the importance of understanding not only the effects of prebiotic compounds but also their underlying mechanisms of action. Whereas various compounds have shown promise in altering microbial composition, comprehensive studies examining both their in vitro and *in vivo* effects, along with detailed mechanistic analyses, are limited. Such integrated approaches are essential for developing effective therapeutic strategies targeting specific beneficial bacteria like *F. prausnitzii*.

The primary aim of this study was to conduct a comprehensive investigation of prebiotic compounds that showed potential for specifically promoting *F. prausnitzii* growth. We employed both in vitro and *in vivo* approaches to validate their effects on gut microbiota composition and conducted mechanistic studies to understand how these compounds selectively enhance *F. prausnitzii* growth. This multi-faceted approach aimed to provide deeper insights into targeted prebiotic strategies for improving gut health.

## Materials and methods

### Bacterial strains and culture conditions

The bacterial strains used in this study were *Faecalibacterium prausnitzii* NCIMB 13872^T^, *F. prausnitzii* JCM 39207, *F. longum* JCM 39211^T^, *F. hattorii* JCM 39210^T^, *Parabacteroides distasonis* JCM 5825^T^, and *P. merdae* JCM 9497^T^. All strains were obtained from their respective culture collections.

*Faecalibacterium* strains were revived and maintained in a Modified Reinforced Clostridial Medium (mRCM). The mRCM was prepared according to the ATCC formulation. The medium was adjusted to pH 6.8 ± 0.2 at 25°C. Cultures were incubated under anoxic conditions (80% N_2_, 10% CO_2_, 10% H_2_) at 37°C [14].

*Parabacteroides* strains were recovered and maintained in GAM (Gifu Anaerobic Medium) broth (Nissui Pharmaceutical Co., Tokyo, Japan) [15]. The cultures were incubated under the same anoxic conditions as *F. prausnitzii* at 37°C.

### Anoxic fecal culture model

The Meiji Institutional Review Board approved the study protocol (approval number: 225).

Fresh fecal samples were collected by Faeces tube, with blade (SARSTEDT AG & Co., Nümbrecht, Germany) from 57 healthy volunteers were immediately homogenized and diluted by 1:10 (w/v) in PreserWell (Fujiwara Seisakusho Co., Ltd., Tokyo, Japan) under anoxic conditions [16]. The diluted samples were then stored at −80°C until further use.

The culture medium used was GAM Semisolid without Dextrose (Nissui Pharmaceutical Co.) via filtration to remove the agar components [15].

For initial screening, fecal samples from six randomly selected volunteers were used to evaluate sixteen saccharides: maltobionic acid (Biosynth Carbosynth, Compton, UK), turanose (Tokyo Chemical Industry Co., Ltd., Tokyo, Japan), leucrose (Apollo Scientific, Stockport, UK), maltotetraose (Toronto Research Chemicals, North York, Canada), and other compounds including lactobionic acid, trehalose, inulin, kestose, laminaran, maltulose, pullulan, curdlan, acacia gum, palatinose, kojibiose, and zymosan (all from FUJIFILM Wako Pure Chemical Corporation). Each compound was added to the medium at a final concentration of 0.3% (w/v).

Following the screening, detailed analyses were conducted using all 57 fecal samples. The prepared medium was supplemented with either maltobionic acid or lactobionic acid at a final concentration of 0.3% (w/v). Sterile water was used as a control. For mechanistic analysis, eight randomly selected fecal samples were used to test D-galacturonic acid monohydrate, D-glucuronic acid, gluconic acid, and D-glucono-1,5-lactone (all from FUJIFILM Wako Pure Chemical Corporation) at a final concentration of 0.3% (w/v).

Diluted fecal samples were inoculated with 1% (v/v) into the prepared medium in deep-well 96-well plates (Thermo Fisher Scientific, Waltham, MA, USA). Cultures were incubated under anoxic conditions at 37°C for 48 h.

### Mono-culture and indirect co-culture experiments

#### Mono-culture experiments with *Faecalibacterium*

Mono-culture experiments were conducted using *Faecalibacterium* strains in 96-well plates. YCFA (yeast extract-casein hydrolysate-fatty acid) medium without sugars was used as the base medium, supplemented with D-glucose, maltobionic acid, or lactobionic acid at a final concentration of 0.3% (w/v).

Overnight cultures of *Faecalibacterium* were inoculated at 0.1% (v/v) into the prepared medium and incubated for 24 h under anoxic conditions.

Following the initial experiments, additional mono-culture studies were conducted to evaluate the growth of *Faecalibacterium* with metabolites produced by *Parabacteroides*. These included L-malic acid, sodium fumarate (Nacalai Tesque, Inc., Kyoto, Japan), L-glutamine, L-tartaric acid, D-galacturonic acid monohydrate, D-glucuronic acid, gluconic acid, and D-glucono-1,5-lactone (all purchased from FUJIFILM Wako Pure Chemical Corporation, Osaka, Japan, unless otherwise noted).

#### Indirect co-culture experiments

Indirect co-culture experiments were performed to investigate potential cross-feeding between *Parabacteroides* and *Faecalibacterium*. The prepared GAM Semisolid without Dextrose medium was supplemented with D-glucose, gluconic acid, maltobionic acid, or lactobionic acid at 0.3% (w/v).

*Parabacteroides* strains were cultured in the prepared media for 24 h under anoxic conditions. After incubation, the culture supernatants were collected by centrifugation and filter-sterilized to remove bacterial cells. Aliquots of cell-free supernatants were stored at −30°C for subsequent metabolomic analysis. The remaining supernatants were inoculated with 0.1% (v/v) of overnight-grown *Faecalibacterium* cultures and incubated for 24 h under anoxic conditions.

Acetate concentrations in *Parabacteroides* culture supernatants were measured using a Cedex Bio HT Analyzer (Roche Diagnostics, Indianapolis, IN, USA) and a Cedex Bio Acetate Kit. The measurements were performed according to the manufacturer's instructions.

#### Growth evaluation

The bacterial growth in both mono-culture and indirect co-culture experiments was assessed by measuring the optical density at 600 nm (OD600) using a Stratus microplate reader (Cerillo, Charlottesville, VA, USA). Growth rates were calculated from the logarithmic growth phase of each culture by linear regression of the natural logarithm of OD600 values against time. The slope of this regression line represents the specific growth rate (μ) in units of h^−1^. Each condition was measured in biological duplicate, and the mean growth rates with standard deviations were calculated.

All experiments were performed in duplicate or triplicate.

### Clinical trial

A single-arm, before-after comparison study was conducted to evaluate the effect of maltobionic acid calcium salt on the gut microbiota. The study was registered in the UMIN Clinical Trials Registry (Trial ID: UMIN000052735).

#### Participants

The participants were recruited from a pool of 49 individuals who showed an increase in the relative abundance of *Faecalibacterium* in fecal cultures (2. Anoxic fecal culture model). Of these, 27 individuals (10 males and 17 females, aged 27 to 58 years, mean age 39.5 y) voluntarily participated in the clinical trial.

The exclusion criteria were as follows:

Participation in other intervention studies within one month prior to screening or plans to participate in such studies during the intervention period.

History of gastric or lower gastrointestinal surgery (excluding hemorrhoid surgery).

Gastrointestinal examinations (e.g. barium radiography, endoscopy) or intestinal cleansing within one month prior to screening or plans to do so during the study period.

Regular use (once a week or more) of medications that could potentially affect the study outcomes, including antibiotics, proton pump inhibitors, diabetes medications (e.g. metformin), laxatives (e.g. magnesium oxide), and bile acid promoters (e.g. ursodeoxycholic acid) within one month before screening, or plans to use such medications during the study period.

#### Intervention

The participants consumed 5 g of maltobionic acid calcium salt (San-ei Sucrochemical Co., Ltd., Aichi, Japan) daily, equivalent to 3 g of maltobionic acid, for two weeks.

#### Sample collection

Fecal samples were collected before and after the intervention period using dedicated sampling kits (FS-0016; Techno Suruga Laboratory Co., Ltd., Shizuoka, Japan) containing a preservation solution and a brush-type collector. The samples were then stored at room temperature.

#### Ethical considerations

The Meiji Institutional Review Board approved the study protocol (approval number: 229). Informed consent was obtained from all participants before enrollment in the study. All participants were healthy volunteers. The study was conducted in accordance with the principles of the Declaration of Helsinki.

### Microbial community analysis

#### DNA extraction

Genomic DNA was extracted from the cultured and fecal samples using the Maxwell RSC PureFood GMO & Authentication Kit (AS1600, Promega, Madison, WI, USA) with some modifications. Briefly, 200 μl of cultured medium or fecal suspension in preservation solution was added to a 2 ml sample tube containing 0.3 g of 0.1 mm zirconia beads. One ml of CATB Buffer was added, and the mixture was vortexed before incubation at 95°C for 15 min in a heat block.

Cell lysis was performed using a FastPrep-24 5G instrument (MP Biomedicals, Santa Ana, CA, USA) with the following settings: speed 5 m/s, 60 s per cycle, and 5 min rest between cycles, for a total of four cycles. The samples were then cooled on ice for 2 min and centrifuged at 16 000 *g*, 4°C for 5 min. The supernatant was transferred to a new 1.5 ml tube. The remaining pellet was re-extracted with 300 μl of CATB Buffer following the same procedure, and the resulting supernatant was combined with the first extract.

A 200 μl aliquot of the combined supernatant was transferred to a new 1.5 ml tube, to which 40 μl of Proteinase K Solution and 20 μl of RNase A (both provided in the kit) were added and gently mixed by vortexing. The mixture was incubated at 70°C for 10 min in a heating block. Subsequently, 300 μl of lysis buffer was added, and the entire sample was loaded onto the Maxwell RSC cartridge.

DNA was eluted in 200 μl of elution buffer using the automated Maxwell RSC instrument according to the manufacturer's instructions. The extracted DNA was quantified and stored at −20°C until further analysis.

#### 16S rRNA gene sequencing and analysis

##### Library preparation and sequencing

The 16S rRNA gene amplicon libraries were prepared following the Illumina 16S Metagenomic Sequencing Library Preparation protocol. The V3-V4 region of the 16S rRNA gene was amplified using the recommended primers. PCR products were purified, indexed, and pooled according to the manufacturer’s instructions. Sequencing was performed on an MiSeq platform using the MiSeq Reagent Kit v3 (600-cycle) (Illumina, San Diego, CA, USA).

##### Sequence data analysis

Sequence data were analyzed using QIIME 2 version 2020.8 [17]. The raw sequencing reads were demultiplexed, quality-filtered, and denoised using the DADA2 plugin [18]. The number of reads for each sample was rarefied to 5000 to ensure an equal sequencing depth across all samples. Amplicon Sequence Variants were clustered and taxonomically classified at the genus level using the Silva 138 database [19]. All subsequent analyses were performed using the genus-level taxonomic data.

#### Quantitative PCR for *Faecalibacterium*

Quantitative PCR (qPCR) was performed to determine the abundance of *Faecalibacterium*. The following primers targeting the *Faecalibacterium* 16S rRNA-encoding gene were used [20]: Forward 5'-GGAGGAAGAAGGTCTTCGG-3′ and Reverse 5'-AATTCCGCCTACCTCTGCACT-3.’

The qPCR reactions were carried out in a total volume of 20 μl, containing 10 μl of PowerUp SYBR Green Master Mix, 0.1 μl each of forward and reverse primers (100 μM), 5.8 μl of nuclease-free water, and 4 μl of template DNA. The reactions were performed on a QuantStudio 3 Real-Time PCR System (Thermo Fisher Scientific) with the following thermal cycling conditions: initial denaturation at 50°C for 2 min and 95°C for 2 min, followed by 40 cycles of 95°C for 15 s and 60°C for 1 min. Dissociation step was performed at the end of the amplification.

For absolute quantification, a standard curve was generated using DNA extracted from a pure culture of *F. prausnitzii* NCIMB 13872^T^. All samples and standards were analyzed in duplicate.

For the additional experiment quantifying *Faecalibacterium* using qPCR, statistical analysis was performed using the Friedman test, followed by pairwise Wilcoxon signed-rank tests. *P* values were adjusted for multiple comparisons using the Benjamini-Hochberg method. Results were considered statistically significant at an adjusted *P* value <0.05.

#### Microbial community analysis of cultured samples

To focus on the analysis of the most abundant and potentially relevant taxa, we limited our statistical tests to the top 15 genera based on their median relative abundance across all samples and conditions.

The relative abundance of specific bacterial genera in the cultured samples were compared across different conditions using non-parametric statistical methods suitable for paired data. Friedman test was performed to determine the presence of any significant differences among the groups. The significance level was set at *P* < .01 to apply a more stringent criterion. Additionally, the effect size (Kendall's W) was calculated to assess the magnitude of differences. When significant differences were indicated by the Friedman test (*P* < .01) and the effect size was moderate or large (Kendall's W > 0.3), post-hoc pairwise comparisons were conducted using Wilcoxon signed-rank tests. To control for multiple comparisons, a more conservative Bonferroni correction method was used instead of the Benjamini-Hochberg procedure.

#### Microbial community analysis of clinical trial fecal samples

As with the cultured samples, we limited our analysis to the top 15 genera based on their median relative abundance across all samples to focus on the most abundant and potentially relevant taxa.

Changes in the relative abundance of bacterial genera between pre- and post-intervention samples were evaluated using the Wilcoxon signed-rank tests. To investigate the factors that might influence responsiveness to the intervention, we performed a correlation analysis. The change in *Faecalibacterium* abundance (post-intervention minus pre-intervention) was correlated with the relative pre-intervention abundance of all other genera using Spearman's rank correlation.

For both Wilcoxon signed-rank tests and Spearman's correlations, *P* values were adjusted for multiple comparisons using the Benjamini-Hochberg method to control for the false discovery rate. The results were considered statistically significant at an adjusted *P* value <0.05.

#### Statistical analysis and visualization

All statistical analyses were performed using R version 4.3.2 (R Core Team, 2023) and RStudio version 2023.06.2; (RStudio Team, 2023). The results were visualized using the ggplot2 package in R.

### Metabolomic analysis

Metabolome analysis was performed by Human Metabolome Technologies, Inc. (Tsuruoka, Yamagata, Japan), using the Basic Scan package.

#### Metabolite extraction

The culture medium samples were centrifuged to remove debris. Subsequently, 80 μl of the supernatant was mixed with 20 μl of Milli-Q water containing internal standards (H3304–1002, HMT). The mixture was then centrifugally filtered via a Millipore 5 kDa cutoff filter (ULTRAFREE MC PLHCC, HMT) at 9100 × *g* and 4°C for 120 min to remove macromolecules. The resulting filtrate was used for subsequent metabolomic analysis.

#### Metabolome analysis

Metabolomic analysis was conducted using capillary electrophoresis time-of-flight mass spectrometry (CE-TOFMS), as previously described methods [21, 22].

A fused silica capillary (50 μm i.d. × 80 cm total length) was used with commercial electrophoresis buffers (H3301–1001 and I3302–1023 for cation and anion analyses, respectively, HMT) as the electrolyte. The mass spectrometer was scanned from m/z 50 to 1000. Peak extraction was performed using the MasterHands automatic integration software (Keio University, Tsuruoka, Yamagata, Japan) to obtain peak information, including m/z, peak area, and migration time (MT) [23].

The signal peaks corresponding to isotopomers, adduct ions, and other product ions of known metabolites were excluded. The remaining peaks were annotated according to the HMT metabolite database based on their m/z values and MTs. Peak areas were normalized to the internal standards and sample amounts to obtain the relative levels of each metabolite.

Principal component analysis (PCA) was performed using HMT's proprietary MATLAB and R programs [24]. The detected metabolites were plotted on metabolic pathway maps using VANTED software [25].

### HPLC–MS/MS analysis of Glucuronic and Galacturonic acids

#### Metabolite extraction

The culture samples were centrifuged at 12000 rpm for 10 min at 4°C, and the supernatant was collected. Protein precipitation was performed using Carrez reagents according to a previously described method [26], where 7.5 μl each of Carrez I and Carrez II solutions were added to 300 μl of sample. After centrifugation at 12000 rpm for 10 min at 4°C, the supernatant was filtered through a ChromaDisc 4A filter (GL Sciences, Tokyo, Japan) into PP vials.

#### UPLC–MS/MS analysis

The analysis was performed using an Intrada Organic Acid column (150 × 2 mm; Imtakt, Kyoto, Japan) maintained at 60°C. The mobile phase consisted of (A) acetonitrile/water/formic acid (10:90:0.1, v/v/v) and (B) acetonitrile/100 mM ammonium formate (10:90, v/v). The flow rate was set at 0.2 ml/min. The gradient elution program was as follows: 0–1 min, 0% B; 1–7 min, linear gradient from 0 to 100% B; 7–10 min, 100% B. The injection volume was 5 μl.

#### Mass spectrometry parameters

Mass spectrometric analysis was performed using a Waters Xevo TQ-S micro system equipped with an electrospray ionization source operating in negative mode. The MS parameters were: capillary voltage, 2.5 kV; cone voltage, 20 V; source temperature, 150°C. Selected ion recording mode was used to monitor m/z 193.06.

#### Chemicals and reagents

All solvents used were LC-MS grade. Formic acid (99%, FUJIFILM Wako Pure Chemical Corporation, Cat. No. 067–04531), ammonium formate (special grade, FUJIFILM Wako Pure Chemical Corporation, Cat. No. 010–03122), and acetonitrile (LC/MS grade, FUJIFILM Wako Pure Chemical Corporation) were used for preparation.

## Results

### Maltobionic and lactobionic acids selectively enhance *Faecalibacterium* abundance in fecal cultures

Initial screening of various saccharides in fecal cultures revealed several compounds that influenced *Faecalibacterium* composition (Supplementary Fig. 1). Among the tested compounds, maltobionic acid and lactobionic acid showed particularly strong effects on *Faecalibacterium* abundance. Based on these promising results, we conducted a more detailed analysis of these two compounds.

**Figure 1:**
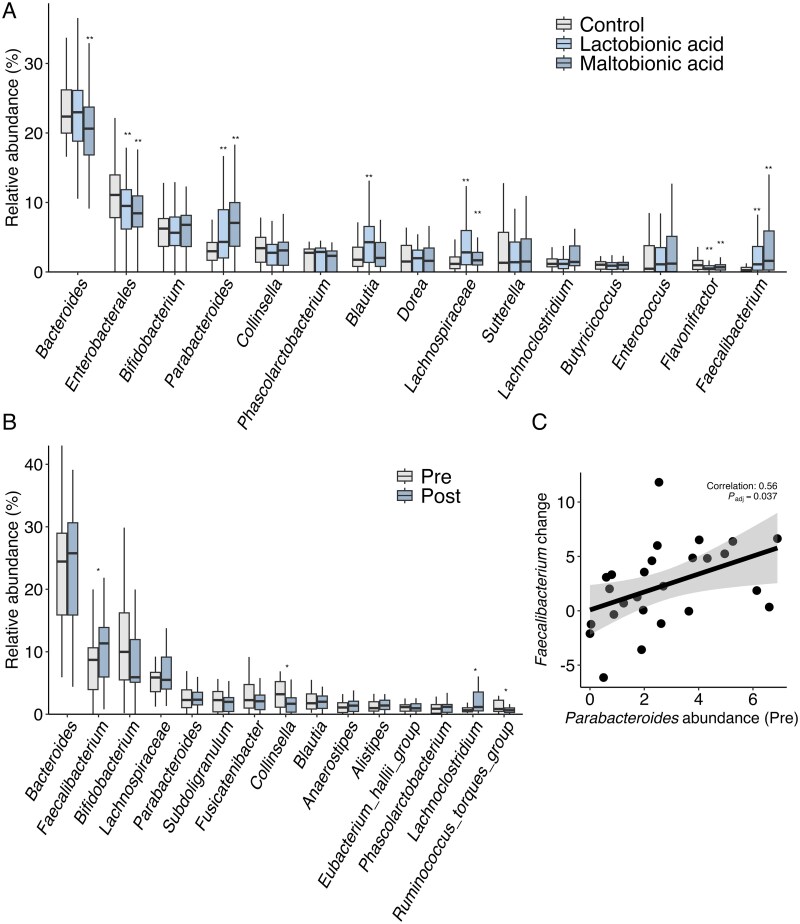
**Effects of maltobionic acid and lactobionic acid on gut microbiota composition in fecal cultures and human subjects.** (**A**) relative abundance of the top 15 bacterial genera in fecal cultures under different conditions. Boxes represent the interquartile range; whiskers extend to 1.5 times the interquartile range, and the line inside the box represents the median. Asterisks indicate significant differences compared to control (^*^^*^P_adj_ < 0.01; Friedman test followed by post-hoc Wilcoxon signed-rank tests with Bonferroni correction). (**B**) changes in the relative abundance of the top 15 bacterial genera before and after maltobionic acid calcium supplementation in the clinical trial. Boxes are represented as in (a). Asterisks indicate significant differences (^*^P_adj_ < 0.05; Wilcoxon signed-rank test with Benjamini-Hochberg correction). (**C**) correlation between the change in *Faecalibacterium* abundance and pre-intervention *Parabacteroides* abundance in the clinical trial. Each point represents an individual participant. The line represents the linear regression fit, and the shaded area represents the 95% confidence interval.

To validate and further characterize the effects of maltobionic and lactobionic acids on gut microbiota composition, we performed comprehensive fecal culture experiments with a larger sample size (Fig. 1A).

Both maltobionic acid and lactobionic acid significantly increased the relative abundance of *Faecalibacterium* compared to that in the control condition (*P_adj_* = 2.56 × 10^−9^ and *P_adj_* = 3.81 × 10^−8^, respectively). No significant difference was observed between the effects of maltobionic and lactobionic acids on *Faecalibacterium* abundance (*P_adj_* = 0.21), suggesting that both compounds had a similar capacity to influence *Faecalibacterium* growth.

Other genera, including *Parabacteroides*, *Bacteroides*, and members of the Lachnospiraceae family, also showed significant changes in response to treatment. These results indicated that maltobionic and lactobionic acids substantially impacted the composition of the gut microbiota in vitro, affecting multiple bacterial taxa.

### Maltobionic acid supplementation increases *Faecalibacterium* abundance in human gut microbiota

Analysis of fecal samples from 27 participants who completed the two-week intervention with maltobionic acid calcium salt supplementation revealed significant changes in gut microbiota composition. Consistent with our in vitro analysis, we concentrated on the top 15 bacterial genera based on median relative abundance.

Wilcoxon signed-rank tests with Benjamini-Hochberg correction for multiple comparisons revealed significant changes in four bacterial genera (*P_adj_* < 0.05). *Faecalibacterium* showed a significant increase following the intervention (*P_adj_* = 0.018), which was consistent with our observations in the fecal culture experiments. Other genera that showed significant changes included *Collinsella* (*P_adj_* = 0.018, decrease), *Lachnoclostridium* (*P_adj_* = 0.028, increase), and *Ruminococcus torques* group (*P_adj_* = 0.018, decrease) (Fig. 1B).

*Bifidobacterium*, a genus often targeted by traditional prebiotics, did not show a significant change (*P_adj_* = 0.19), suggesting that maltobionic acid may have a more selective effect on certain bacterial groups, particularly *Faecalibacterium*. Additionally, we found a significant positive correlation between the changes in *Faecalibacterium* and the pre-intervention abundance of *Parabacteroides* (Spearman's ρ = 0.56, *P_adj_* = 0.037) (Fig. 1C). After adjusting for multiple comparisons, *Parabacteroides* was the only genus that showed a significant correlation with the changes in *Faecalibacterium* abundance, highlighting its potential role in the observed prebiotic effect.

### Investigation of direct growth promotion and cross-feeding mechanisms

#### *Faecalibacterium* species cannot directly utilize maltobionic or lactobionic acid

To investigate the direct effects of maltobionic acid and lactobionic acid on *Faecalibacterium* growth, we conducted mono-culture experiments using three *Faecalibacterium* species: *F. prausnitzii* NCIMB 13872^T^, *F. longum* JCM 39211^T^, and *F. hattorii* JCM 39210^T^.

**Figure 2:**
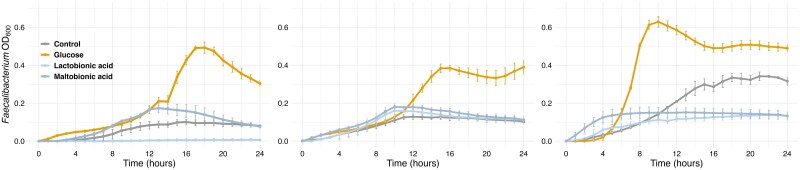
**Growth curves of *Faecalibacterium* species in mono-culture under conditions involving different substrates**. Growth curves of three *Faecalibacterium* species in mono-culture under conditions involving different substrates (control, glucose, Lactobionic acid, and Maltobionic acid). The OD_600_ was measured over 24 h. left panel: *F. Prausnitzii* NCIMB 13872^T^, middle panel: *F. Longum* JCM 39211^T^, right panel: *F. Hattori* JCM 39210^T^. Error bars represent the standard deviation from triplicate experiments.

Our results showed that all three *Faecalibacterium* species exhibited significant growth when glucose was added to the medium (Fig. 2). In contrast, neither maltobionic nor lactobionic acids promoted the growth of any *Faecalibacterium* species when added directly to the culture medium. The growth curves for these two conditions were similar to those of the control.

These findings suggest that *Faecalibacterium* species are unable to directly utilize maltobionic acid or lactobionic acid as the primary carbon source for growth.

#### Metabolism of *Parabacteroides* enhances the growth of *Faecalibacterium* via cross-feeding

Given the correlation between pre-intervention *Parabacteroides* abundance and the increase in *Faecalibacterium* observed in our clinical trial, as well as the significant increase in *Parabacteroides* in our fecal culture experiments, we investigated the potential cross-feeding mechanisms between these genera. We focused on two *Parabacteroides* species, *P. distasonis* JCM 5825^T^, and *P. merdae* JCM 9497^T^, which represent the majority of *Parabacteroides* in the human gut [27]. For *Faecalibacterium*, we used *F. prausnitzii* NCIMB 13872^T^ and *F. prausnitzii* JCM 39207 because *F. prausnitzii* is the primary species of *Faecalibacterium* in humans and has been extensively studied for its various beneficial effects [28].

**Figure 3:**
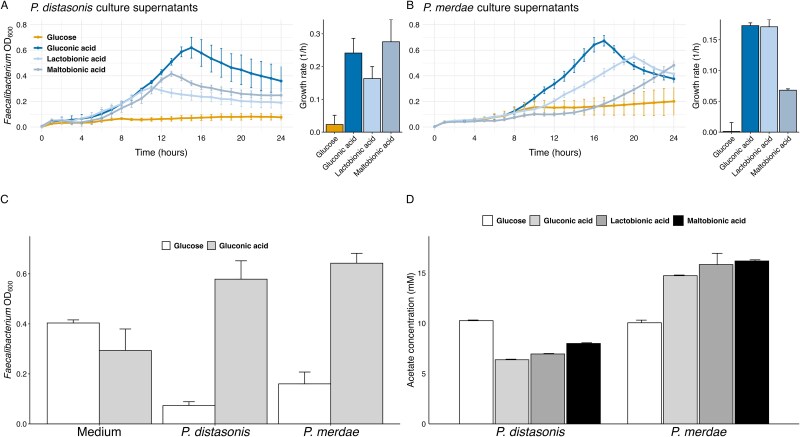
**Cross-feeding between *Parabacteroides* and *F. Prausnitzii* in indirect co-culture experiments**. (**A**) growth curves of *F. Prausnitzii* NCIMB 13872^T^ in *P. Distasonis* JCM 5825^T^ culture supernatants with different carbon sources. Growth rates were calculated during the logarithmic growth phase (6–12 h). Error bars represent standard deviation from biological duplicates. (**B**) growth curves and growth rates of *F. Prausnitzii* NCIMB 13872^T^ in *P. Merdae* JCM 9497^T^ culture supernatants. Growth rates were calculated during the logarithmic growth phase (10–16 h). Error bars represent standard deviation from biological duplicates. (**C**) OD600 values of *F. Prausnitzii* NCIMB 13872^T^ after 16 h of growth in different media conditions. “medium” indicates direct supplementation with glucose (white bars) or gluconic acid (gray bars), whereas “*P. Distasonis*” and “*P. Merdae*” represent growth in respective *Parabacteroides* culture supernatants supplemented with glucose or gluconic acid. Error bars represent standard deviation from duplicate experiments. (**D**) acetate concentrations in *Parabacteroides* culture supernatants under different conditions. Error bars represent standard deviation from triplicate experiments.

We cultured the *Parabacteroides* strains in a medium supplemented with glucose, gluconic acid, maltobionic acid, or lactobionic acid. The resulting culture supernatants were used to culture *F. prausnitzii* strains. Both *F. prausnitzii* strains showed enhanced growth in supernatants from *Parabacteroides* cultures supplemented with gluconic acid, maltobionic acid, or lactobionic acid, compared to those supplemented with glucose (Fig. 3A,B and Supplementary Fig. 2A). This suggests that *Parabacteroides* may metabolize these compounds into products that can be utilized by *F. prausnitzii*.

Direct supplementation of gluconic acid to the growth medium did not significantly enhance *F. prausnitzii* growth (Fig. 3C and Supplementary Fig. 2B). These results demonstrate that the metabolic conversion of gluconic acid by *Parabacteroides* is essential for enhancing *F. prausnitzii* growth, rather than the direct utilization of gluconic acid itself. This finding suggests a specific cross-feeding pathway where *Parabacteroides* converts gluconic acid into metabolites that are particularly beneficial for *F. prausnitzii* growth.

As *F. prausnitzii* is known to increase in abundance with acetate supplementation [3] and *Parabacteroides* are known acetate-producing bacteria [29], we investigated the potential role of acetate in this cross-feeding mechanism. We measured the acetate concentrations in the culture supernatants of *Parabacteroides* grown on various substrates. However, acetate production patterns did not consistently align with the growth patterns observed in *F. prausnitzii* (Fig. 3D), suggesting that other metabolites may play a more significant role in the observed cross-feeding.

#### Gluconic acid alters *Parabacteroides* metabolite profile, revealing potential cross-feeding substrates

To elucidate the metabolic changes induced by gluconic acid in *Parabacteroides* species, we performed a comprehensive metabolomic analysis of the culture supernatants from *P. distasonis* JCM 5825^T^ and *P. merdae* JCM 9497^T^ grown with and without gluconic acid supplementation.

**Figure 4:**
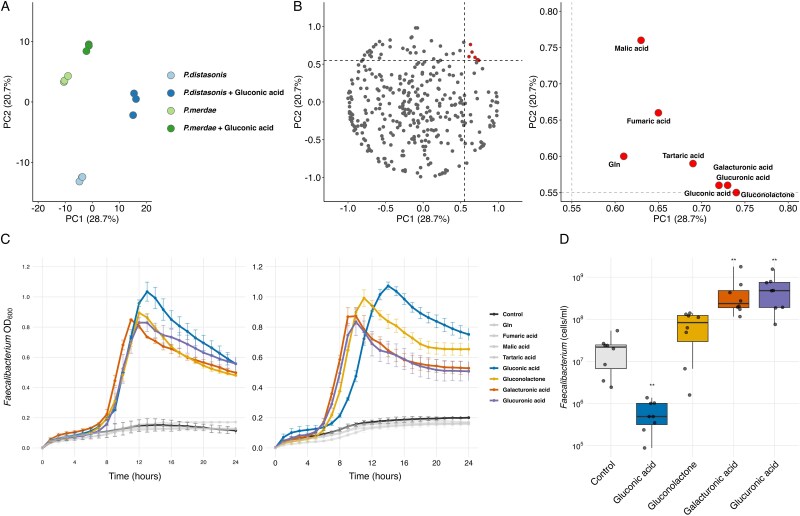
**Metabolomic analysis and verification of candidate metabolites**. (**A**) PCA plot of metabolite profiles from *Parabacteroides* cultures under different conditions. (**B**) scatter plots showing the loadings of metabolites on PC1 and PC2. Left panel: All detected metabolites. Right panel: Metabolites with PC1 and PC2 loadings >0.55 are highlighted and labeled. (**C**) growth curves of *F. Prausnitzii* NCIMB 13872^T^ (left panel) and JCM 39207 (right panel) in the mono-culture supplemented with selected metabolites identified from the metabolomic analysis. OD600 was measured over 24 h. Error bars represent the standard deviation from duplicate experiments. (**D**) relative abundance of *Faecalibacterium* in fecal cultures from eight donors supplemented with selected metabolites identified from the metabolomic analysis. Box plots show the median and interquartile range, and whiskers extend to 1.5 times the interquartile range. Individual data points are overlaid. Asterisks indicate significant differences compared to the control condition (^*^^*^P_adj_ < 0.01; Wilcoxon signed-rank test with Benjamini-Hochberg correction).

PCA of the metabolomic data revealed a distinct clustering of samples based on both the bacterial species and the presence of gluconic acid (Fig. 4A). The first principal component (PC1) and the second principal component (PC2) showed positive shifts in cultures supplemented with gluconic acid. This indicated that gluconic acid supplementation led to significant changes in the metabolic profiles of both *Parabacteroides* species.

To identify the metabolites that contributed the most significantly to these differences, we examined the loadings of individual compounds on both PC1 and PC2 (Fig. 4B). We focused on metabolites with loadings greater than 0.55 on both PC1 and PC2, as these compounds are likely to be most strongly associated with the metabolic changes induced by gluconic acid. This analysis revealed several key metabolites including malic acid, fumaric acid, glutamine, tartaric acid, galacturonic acid, glucuronic acid, and gluconolactone.

These findings suggest that *Parabacteroides* metabolize gluconic acid into various organic acids and related compounds.

The complete metabolomic dataset, including all detected metabolites and their relative abundance across all conditions and replicates, is provided in Supplementary Table S1.

#### Glucuronic acid is a key growth factor for *Faecalibacterium* in complex microbial communities

Following metabolomic analysis, we investigated the direct effects of the identified metabolites on *F. prausnitzii* growth. We first evaluated the growth-promoting potential of these compounds using pure cultures of the two *F. prausnitzii* strains and measured the OD as an indicator of growth (Fig. 4C).

Our initial screening revealed that among the metabolites identified, gluconic acid, glucono-δ-lactone, galacturonic acid, and glucuronic acid significantly enhanced the growth of both *F. prausnitzii* strains. This suggests that the compounds produced by *Parabacteroides* during gluconic acid metabolism may serve as key substrates for *F. prausnitzii*.

To validate these results in a more complex microbial environment, we conducted a series of experiments using fecal cultures. Eight fecal samples were randomly selected and supplemented with four growth-promoting compounds identified in the pure culture experiments. The abundance of *F. prausnitzii* was quantified using qPCR targeting the 16S rRNA-encoding gene (Fig. 4D).

Among the tested compounds, glucuronic acid and galacturonic acid consistently increased the abundance of *F. prausnitzii* in all fecal samples. This observation corroborates our findings from the pure culture experiments and suggests that these compounds play a crucial role in supporting *F. prausnitzii* growth within the complex gut microbial community.

To further validate the metabolic conversion of gluconic acid by *Parabacteroides*, we performed HPLC-MS/MS analysis of culture supernatants. The chromatographic separation of glucuronic acid and galacturonic acid standards showed distinct peaks at retention times of 2.64 and 2.95 min, respectively (Supplementary Fig. 3). When *P. distasonis* was cultured in medium containing gluconic acid, we detected a clear peak corresponding to glucuronic acid. In contrast, this peak was absent in the culture supernatant without gluconic acid supplementation. These results provide direct evidence that *P. distasonis* converts gluconic acid to glucuronic acid, which serves as a key growth factor for *F. prausnitzii*.

## Discussion

This study presents two significant findings that advance our understanding of prebiotic effects and microbial interactions in the gut ecosystem. Our first key finding, which states that maltobionic acid specifically enhances the growth of *Faecalibacterium* both in vitro and *in vivo*, represents a significant advancement in targeted prebiotic approaches. The consistency of *Faecalibacterium* enrichment across laboratory cultures and the effects observed in human intervention studies underscores the robustness of this effect. Our results showed that maltobionic acid did not promote the growth of *Bifidobacterium*, a genus commonly targeted by traditional prebiotics, highlighting its specificity [30]. This selective stimulation of *Faecalibacterium* is particularly important, given its association with various health outcomes and its potential role in maintaining gut homeostasis [31].

Our second key finding elucidates a cross-feeding mechanism underlying the prebiotic effects of maltobionic acid, revealing a complex interspecies metabolic relationship in the gut microbiome. We demonstrated that *Parabacteroides* converted the gluconic acid moiety of gluconic acid-containing oligosaccharides into glucuronic acid, which, in turn, promoted the growth of *Faecalibacterium*. This finding is particularly intriguing given the relatively small genome size of *Parabacteroides* and its rich capacity for sugar utilization [27, 32].

The stereochemistry of these compounds provides important insights into the metabolic pathways involved. The conversion of gluconic acid into glucuronic acid is stereochemically feasible because both compounds have the same configuration at C4 (R configuration). This transformation has been observed during kombucha fermentation [33], suggesting that it can potentially occur in other microbial systems.

The specific role of glucuronic acid in *Faecalibacterium* metabolism builds upon previous research demonstrating this organism's ability to utilize glucuronic acid [31]. Our study advances this understanding by showing that this metabolic capability remains effective in complex microbial communities despite potential competition from other bacteria.

Although glucuronic acid production in the gut could potentially interact with Toll-like receptor 4 and influence inflammatory responses [34], our findings suggest that such concerns may be minimal in this context. The efficient utilization of glucuronic acid by *Faecalibacterium*, as demonstrated in our study, indicates that this metabolite is rapidly consumed once produced. This active bacterial consumption likely prevents significant accumulation of free glucuronic acid in the gut environment.

Our findings highlight a crucial distinction between the prebiotic effects of gluconic acid and gluconic acid-containing oligosaccharides and shed light on the unique delivery mechanism of glucuronic acid through gluconic acid-containing oligosaccharides. Although gluconic acid has been reported to be a prebiotic [35], our fecal culture experiments showed that it did not significantly increase *Faecalibacterium* abundance. In contrast, our study demonstrated that gluconic acid-containing oligosaccharides were primarily metabolized by *Parabacteroides*, leading to the production of glucuronic acid and a more pronounced growth promotion of *Faecalibacterium*. This differential metabolism explains our observation that the metabolites produced by *Parabacteroides* from gluconic acid-containing compounds enhanced *Faecalibacterium* growth more effectively in co-culture experiments than direct gluconic acid supplementation.

The implications of our findings extend far beyond the scope of this study and offer new perspectives for prebiotic research and potential therapeutic applications. By providing an approach for modulating *Faecalibacterium* levels, our research opens new avenues for the prevention and management of various diseases associated with this beneficial bacterium [36]. Our study highlights the value of in vitro screening methods and the importance of evaluating cross-feeding mechanisms in prebiotics research.

Pre-intervention microbiome profiling showed potential for predicting prebiotic intervention efficacy [37]. Our clinical trial results demonstrated a significant correlation between pre-intervention *Parabacteroides* abundance and increased *Faecalibacterium* following maltobionic acid supplementation, indicating possibilities for personalized nutritional approaches.

It is important to acknowledge several limitations of our study. The detailed molecular mechanisms underlying the conversion of gluconic acid to glucuronic acid by *Parabacteroides* and its utilization by *Faecalibacterium* remain to be fully elucidated. The specific enzymatic pathways involved warrant further investigation using techniques such as transcriptomics, proteomics, and metabolic flux analyses [38]. The technical challenges in genetic manipulation of anaerobic gut bacteria, including *Parabacteroides*, currently limit our ability to fully characterize these pathways.

The clinical study had several limitations. The single-arm design and two-week duration restricted our ability to exclude placebo effects and assess long-term outcomes. Future research should include randomized, placebo-controlled trials with larger and more diverse populations, extended intervention periods, and follow-up measurements to evaluate the sustainability of these prebiotic effects. Additionally, whereas our focused analysis of the top genera effectively demonstrated the specificity of our intervention, future studies using metagenomic sequencing could provide valuable species-level resolution and broader ecological insights.

The variability in *Faecalibacterium* responses among participants in our clinical trial highlights the complex nature of host–microbe interactions and the potential influence of individual factors on prebiotic efficacy [39]. The potential competition for glucuronic acid between *Faecalibacterium* and other gut microbes, and how this competition influences the observed prebiotic effects, remain to be explored. Understanding these aspects is crucial for optimizing the use of gluconic acid-containing oligosaccharides as targeted prebiotics and for predicting their efficacy in different microbial contexts [40]. Whereas our study demonstrated consistent effects across multiple *Faecalibacterium* strains, we acknowledge the growing diversity within this genus as new species continue to be discovered. Further investigation of species-specific responses to these prebiotic compounds and comprehensive metagenomic analyses will be valuable for understanding the broader applicability of this approach across the *Faecalibacterium* genus.

In conclusion, our study provides compelling evidence for the prebiotic effects of maltobionic acid on *Faecalibacterium* and elucidates a cross-feeding mechanism involving *Parabacteroides*. These findings not only contribute to our understanding of microbial interactions in the gut but also open up new possibilities for targeted modulation of the gut microbiome. Future research should focus on characterizing the molecular mechanisms underlying this cross-feeding relationship and exploring the potential therapeutic applications of maltobionic acid in various health contexts.

## Supplementary Material

Supplementary_Figures_wraf027

Supplementary_Table_wraf027

## Data Availability

The raw sequencing data generated in this study have been deposited in the DDBJ Sequence Read Archive under the accession number PRJDB18600 and PRJDB18601.
