# Supplementary material for: Targeted prebiotic application of gluconic acid-containing oligosaccharides promotes Faecalibacterium growth through microbial cross-feeding networks
Source: ISME J. 2025 Feb 12;19(1):wraf027. doi: 10.1093/ismejo/wraf027 (PMC11922316; doi:10.1093/ismejo/wraf027)
Supplement: Supplementary_Figures_wraf027 [file supplementary_figures_wraf027.pdf]

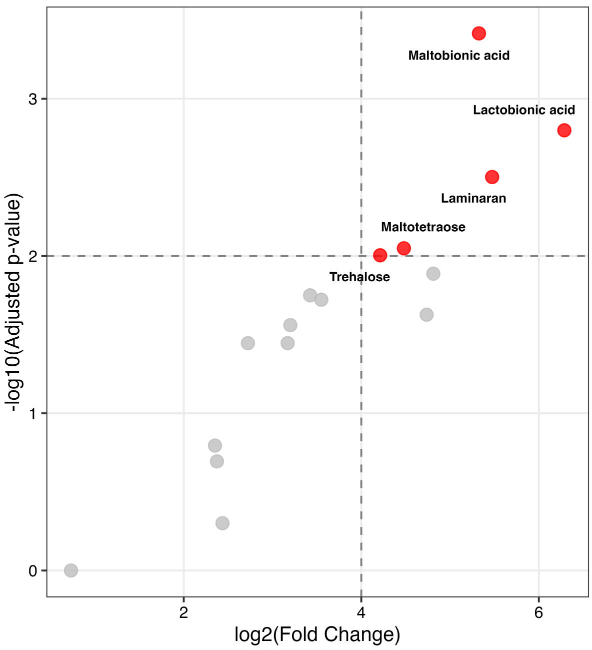

Supplementary Figure 1. Volcano plot showing changes in *Faecalibacterium* in response to various saccharides

The plot displays the relationship between statistical significance (-log10 of Bonferroni-adjusted *P* values) and magnitude of change (log2 fold change) compared to the control condition. Each point represents a different saccharide (n=6 per group). After confirming normality using Shapiro-Wilk test, paired t-test or Wilcoxon signed-rank test was performed, followed by Bonferroni correction for multiple comparisons. Red dots indicate significant increase ($P_{adj} < 0.01$).

A   B

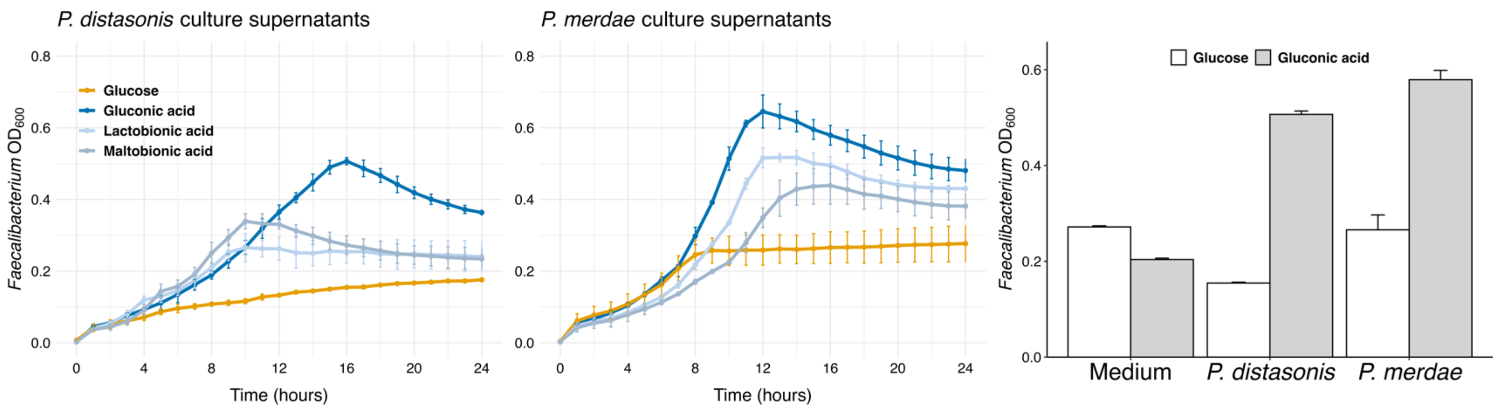

Supplementary Figure 2. Confirmation of cross-feeding between *Parabacteroides* and *F. prausnitzii* JCM 39207 in indirect co-culture experiments

A Growth curves of *F. prausnitzii* JCM 39207 in *Parabacteroides* culture supernatants under different conditions. Left panel: *F. prausnitzii* JCM 39207 in *P. distasonis* JCM 5825^T supernatant, right panel: *F. prausnitzii* JCM 39207 in *P. merdae* JCM 9497^T supernatant. Error bars represent standard deviation from duplicate experiments.

B OD600 values of *F. prausnitzii* NCIMB JCM 39207 after 16 h of growth in different media conditions. "Medium" indicates direct supplementation with glucose (white bars) or gluconic acid (gray bars), while "*P. distasonis*" and "*P. merdae*" represent growth in respective *Parabacteroides* culture supernatants supplemented with glucose or gluconic acid. Error bars represent standard deviation from triplicate experiments.

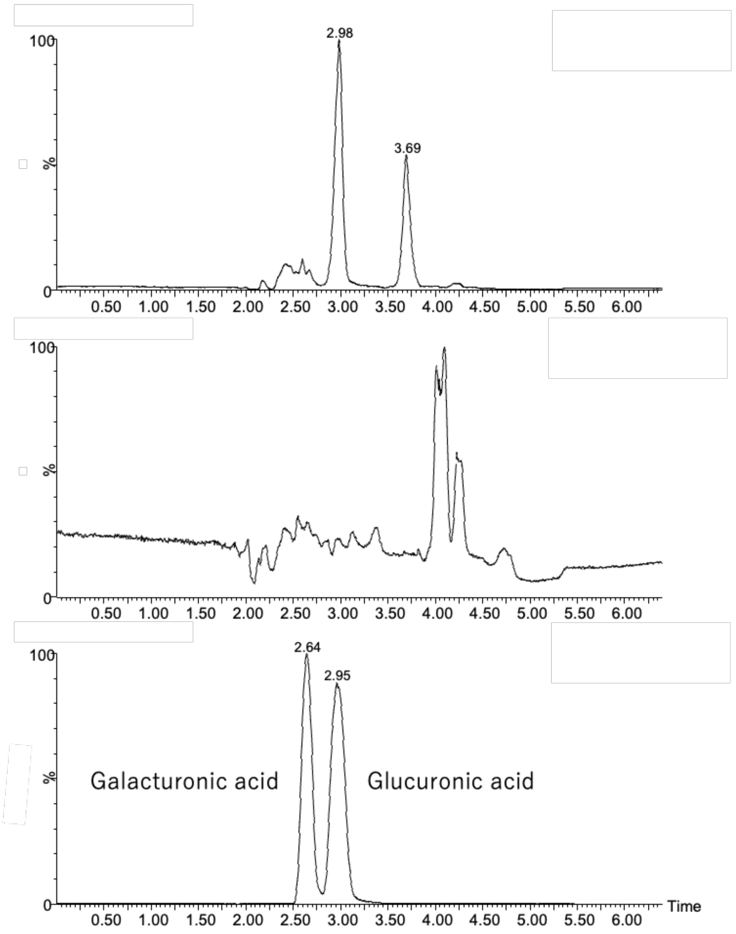

Supplementary Figure 3. LC-MS/MS analysis of gluconic acid metabolism by *P. distasonis*

Representative chromatograms showing the presence of glucuronic acid and galacturonic acid. Top: Culture supernatant of *P. distasonis* grown in medium containing gluconic acid. Middle: Culture supernatant of *P. distasonis* grown in medium without gluconic acid. Bottom: Standard mixture of glucuronic acid and galacturonic acid.
